# Development of Magnetic Lateral Flow and Direct Competitive Immunoassays for Sensitive and Specific Detection of Halosulfuron-Methyl Using a Novel Hapten and Monoclonal Antibody

**DOI:** 10.3390/foods12142764

**Published:** 2023-07-20

**Authors:** Ying Ying, Xueyan Cui, Hui Li, Lingyi Pan, Ting Luo, Zhen Cao, Jing Wang

**Affiliations:** 1 Institute of Quality Standards and Testing Technology for Agro-Products, Key Laboratory of Agro-Product Quality and Safety, Chinese Academy of Agricultural Sciences, Ministry of Agriculture, Beijing 100081, China; yingying@caas.cn (Y.Y.); cxy19910411@163.com (X.C.); urhaan@163.com (L.P.); luoting074@163.com (T.L.); w_jing2001@126.com (J.W.); 2 Institute of Quality Standards and Testing Technology for Agro-Products, Chinese Academy of Agricultural Sciences, Beijing 100081, China

**Keywords:** halosulfuron-methyl, monoclonal antibody, magnetic lateral flow immunoassay

## Abstract

Halosulfuron-methyl (HM) is widely used for the removal of noxious weeds in corn, sugarcane, wheat, rice, and tomato fields. Despite its high efficiency and low toxicity, drift to nontarget crops and leaching of its metabolites to groundwater pose potential risks. Considering the instability of HM, the pyrazole sulfonamide of HM was used to generate a hapten and antigen to raise a high-quality monoclonal antibody (Mab, designated 1A91H11) against HM. A direct competitive immunoassay (dcELISA) using Mab 1A91H11 achieved a half-maximal inhibitory concentration (IC50) of 1.5 × 10^−3^ mg/kg and a linear range of 0.7 × 10^−3^ mg/kg–10.7 × 10^−3^ mg/kg, which was 10 times more sensitive than a comparable indirect competitive ELISA (icELISA) and more simple to operate. A spiking recovery experiment performed in tomato and maize matrices with 0.01, 0.05, and 0.1 mg/kg HM had average recoveries within 78.9–87.9% and 103.0–107.4% and coefficients of variation from 1.1–6.8% and 2.7–6.4% in tomato and maize, respectively. In addition, a magnetic lateral flow immunoassay (MLFIA) was developed for quantitative detection of low concentrations of HM in paddy water. Compared with dcELISA, the MLFIA exhibited 3.3- to 50-fold higher sensitivity (IC50 0.21 × 10^−3^ mg/kg). The average recovery and RSD of the developed MLFIA ranged from 81.5 to 92.5% and 5.4 to 9.7%. The results of this study demonstrated that the developed dcELISA and MLFIA are suitable for rapid detection of HM residues in tomato and maize matrices and paddy water, respectively, with acceptable accuracy and precision.

## 1. Introduction

Due to their high efficiency and low toxicity [1,2,3], sulfonylurea-based herbicides are widely used to remove noxious weeds in maize, cereal, sugarcane, and tomato fields. HM (a sulfonylurea) is the active ingredient in many registered products in China. Currently, there are 14 registered manufacturers of HM pesticides in China, with a total of 22 registered formulations. These formulations are mainly registered for use in 10 crop fields, including winter wheat fields, tomato fields, sugarcane fields, sorghum fields, upland direct-seeded rice fields, paddy fields (direct-seeded), transplanted rice fields, summer corn fields, wheat fields, and corn fields (source: China Pesticide Information Network). The excessive or repeated use of herbicides without following proper scientific methods for weed control can also lead to serious phytotoxicity, directly impacting crop yields. Although HM is metabolized quickly in target crops, its metabolites easily leach to deep layers of the water table with rainwater and irrigation water, causing pollution of water resources, which can endanger human health. In addition, HM residues in water and soil have caused damage to nontarget crops such as soybean [4]. The potential impact of HM metabolites on other nontarget crops and aquatic ecosystems is still not fully understood.

Monitoring of HM and other sulfonylureas in food samples is typically accomplished using instrumental methods, such as liquid chromatography-tandem mass spectrometry (LC-MS/MS) [2,5], capillary electrophoresis (CE)-MS/MS [3], and ultra-pressure liquid chromatography (UPLC) [6]. While these methods are sensitive and accurate, they are time intensive and require operation by specialized personnel. Immunoassay, being a widely utilized method in the rapid detection of pesticides, small molecules, environmental pollutants, viruses, and hormones, offers certain advantages over instrumental analytical methods in terms of simplicity, speed, and portability. The basis of immunoassay lies in the design and preparation of highly specific haptens and antibodies, as well as the selection of appropriate immunodetection modes.

Hapten design considerations include the position of the active site group and the length of the connecting spacer arm, as well as the stability and solubility of the hapten [7,8,9,10]. Due to the instability of sulfonylureas, a common strategy for hapten synthesis involves adding a functional spacer arm to a stable metabolite of sulfonylurea [11,12,13].

For instance, the addition of a succinic acid spacer arm to stable metabolites of triasulfuron, metsulfuron-methyl, and chlorimuron-ethyl resulted in the production of monoclonal antibodies (mAbs) with enhanced sensitivity [11,12,13]. These antibodies were then applied in the development of highly sensitive indirect competitive ELISAs for target molecule detection. In a specific study, Schlaeppi et al. [11] prepared two triasulfuron haptens, one with a functional aminoalkyl group added to the triazine ring and the other with a functional succinic acid spacer arm added to the chloroethoxy sulfonamide moiety. The latter hapten generated a more sensitive mAb for triasulfuron. Similarly, Welzig et al. [12] generated metsulfuron-methyl rabbit antibodies using a hapten synthesized by adding a succinic acid C3-spacer arm to a phenylsulfonyl derivative of metsulfuron-methyl. Zhao et al. [13] included a succinic acid spacer arm in their chlorimuron-ethyl hapten, specifically added to the ethoxycarbonyl phenyl sulfonamide of its metabolite. This hapten enabled the generation of a highly sensitive mAb to chlorimuron-ethyl.

Due to the presence of the sulfonylurea bridge in the molecular structure of HM, the molecule has poor stability, resulting in high cost and low success rate in synthesizing the complete HM molecule as a hapten. This limitation has hindered the development of HM antibodies and the establishment of immunoassay. Therefore, the design of an appropriate hapten is crucial [14]. However, given its hydrolysis to a pyrazole-sulfonamide and pyrimidine amine, strategies similar to those mentioned above are expected to be needed for effective HM hapten design.

Magnetic nanoparticles (MNPs) have good stability, high operability, and excellent biocompatibility, making them broadly applicable to food safety detection, environmental treatments, biological medicine, etc. MNPs are generally employed in analyte separations and enrichment, as drug carriers [15,16], and as signal probes in magnetic lateral flow strips [17,18,19]. Due to their high separation and enrichment efficiency, a magnetic lateral flow immunoassay could provide high sensitivity detection of HM in water.

In this study, haptens of stable metabolites of HM with linkers tethered at alternative sites of the metabolite’s structure were used to raise specific monoclonal antibodies to HM. The resulting monoclonal antibodies were used to establish a highly sensitive dcELISA for rapid detection of residual HM in registered food matrices, including tomato and maize. Additionally, we developed a sensitive lateral flow immunoassay based on 50 nm MNPs for the detection of HM in rice paddy water. These findings have significant implications for the efficient and accurate detection of HM residues and contribute to the development of safer agricultural practices.

## 2. Materials and Methods

### 2.1. Reagents, Buffers, and Instruments

Bovine serum albumin (BSA); ovalbumin (OVA); horseradish peroxidase (HRP); complete and incomplete Freud’s adjuvant; poly-ethylene glycol (PEG, Mw = 1450); hypoxanthine, aminopterin, and thymidine (HAT) medium supplements; cell-freezing medium; dimethyl sulfoxide (DMSO); N-hydroxysulfosuccinimide sodium salt (sulfo-NHS); 1-(3-dimethylaminopropyl)-3-ethylcarbodiimide hydrochloride (EDC); o-phenylenediamine (OPD); and mouse monoclonal antibody isotyping reagents were purchased from Sigma-Aldrich (St. Louis, MO, USA). Succinic anhydride and 5-(aminosulfonyl)-3-chloro-1-methyl-1H-pyrazol, the 50 nm carboxyl magnetic beads (10 mg/mL), and 2-(N-morpholino) ethanesulfonic acid (MES) were from Shanghai yuanye Bio-Technology Co., Ltd. (Shanghai, China). Raw tomato and maize samples were obtained from the Nankou Experimental Station of the Chinese Academy of Agricultural Sciences (Beijing, China), where herbicide treatments were not applied during the cultivation.

Buffers used in this study were made as previously described by Cui et al. [1]. Phosphate-buffered saline (PBS, 0.01 M, pH 7.4), carbonate-buffered saline (cbuffer; 0.5 M, pH 9.6), washing buffer (PBS containing 0.1% Tween-20), sample diluting buffer (PBSTG, PBS containing 0.1% Tween-20 and 0.5% gelatin), and substrate buffer (pH 7.4, 0.01 M citrate–phosphate) were used in sample dilution and ELISA experiments. The 10 mL substrate solution, containing 20 mg OPD and 4 μL of 30% hydrogen peroxide, was prepared freshly before use.

### 2.2. Synthesis of the HM Hapten

**Figure 1:**
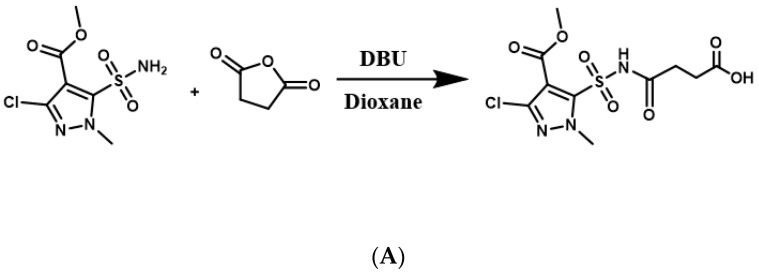

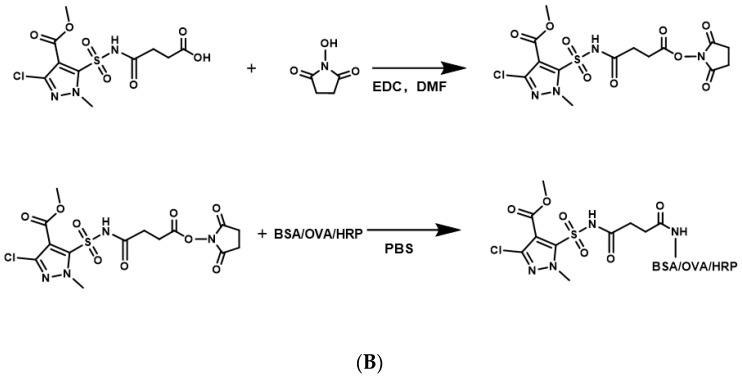
Schematics of HM hapten synthesis (**A**) and HM antigen preparation (**B**).

According to the synthetic procedure in Figure 1A, the HM hapten was synthesized by reacting pyrazole sulfonamide (a HM metabolite) and succinic anhydride. A solution of 5-(aminosulfonyl)-3-chloro-1-methyl-1H- pyrazol (1 g, 4 mmol) and succinic anhydride (0.4 g, 4 mmol) was slowly treated with DBU, and the mixture was stirred at room temperature for 3 h. The reaction mixture was acidified to pH 2 with 2 M hydrochloric acid and extracted three times with 50 mL ethyl acetate. The organic layers were dried over anhydrous Na_2_SO_4_, filtered, and concentrated. The buffer residues were purified with a chromatographic silica gel column using dichloromethane/methyl alcohol (10:1) as the eluent, which yielded HM hapten (0.9 g, 66.6%). The high-resolution mass spectrometry (HRMS) and nuclear magnetic resonance (NMR) spectra of the HM hapten are shown in Figures S1 and S2. HRMS was conducted using a quadrupole time-of-flight mass spectrometer (QTOF MS), and the accurate mass [M-H]^−^ = 352.0019 was detected, which corresponded to C_10_H_12_ClN_3_O_7_S, which has an exact mass of [M-H]^−^ = 352.0006. ^1^H NMR (400 MHz, DMSO) resulted in the following shifts: *δ* 12.32 (b2, H, COOH), *δ* 4.13 (s, 3H, NMe), 3.84 (s, 3H, OMe), 2.53 (dd, *J* = 12.6, 6.3 Hz, 2H, CH2), and 2.42 (t, *J* = 6.1 Hz, 2H, CH2); ^13^C NMR (400 MHz, DMSO): δ 174.36, 134.69, 129.27, 121.91, 121.71, 119.75, 87.75, 27.77, 25.72, and 24.17 ppm.

### 2.3. Preparation of the Protein-Hapten Conjugate

The HM hapten was conjugated with carrier proteins, bovine serum albumin (BSA), ovalbumin (OVA), and horseradish peroxidase (HRP) using the carbodiimide cross-linker method as previously described [20]. Hapten to protein conjugation was carried out following the synthetic route illustrated in Figure 1B. Briefly, the HM hapten, EDC, and sulfo-NHS were dissolved in 500 μL DMSO at a ratio of n(hapten):n(EDC):n(sulfo-NHS) = 1:1.25:1.25 by stirring for 4 h at room temperature. The reaction mixture was then centrifugated at 8452× *g* for 5 min to remove the precipitate. The activated hapten was added dropwise to the protein solution (0.01 M PBS) at a ratio of n(protein):n(hapten) = 1:30 and stirred at 4 °C for 4 h. The mixture was dialyzed against 4 × 4 L PBS at 4 °C over 20 h. After dialysis, BSA-hapten, OVA-hapten, and HRP hapten conjugates were stored at −20 °C. The molar ratio of the protein-hapten was tested on a Bruker Autoflex III matrix-assisted laser desorption/ionization time of flight mass spectrometry (MALDI-TOF-MS; Bruker Corporation, Billerica, MA, USA).

### 2.4. Immunization and Monoclonal Antibody Production

Animal immunization, cell fusion, and monoclonal antibody production protocols were the same as was previously described [20,21,22]. The ethical approval for our animal experiments was granted by the Experimental Animal Welfare and Ethical Committee of Institute of Quality Standards and Testing Technology for Agro-Products, Chinese Academy of Agricultural Sciences (IQSTAP-2021-05). Female BALB/C mice (6–8 weeks old) were immunized with the BSA-hapten conjugate by intraperitoneal and subcutaneous injection. A 100 μg amount of the BSA-hapten conjugate was dissolved in 100 μL PBS and emulsified with 100 μL complete Freund’s adjuvant. Subsequent doses were given using incomplete Freund’s adjuvant every two weeks. The antisera were collected after the third injection to test the titer and target recognition ability of the antibody by checkboard icELISA. Positive mouse spleen cells were fused with SP2/0 myeloma cells at a ratio of 10:1 using PEG with MW = 1450. The fused cells were cultured in HAT selection medium. Ten days after fusion, cell supernatants were screened for their response to HM using icELISA. Individuals exhibiting a strong positive response to HM and high levels of inhibition were isolated using the limiting dilution method. After one or two subclones, the monoclonal hybridoma cells were selected and expanded in a 6-well cell plate. Next, 106 of the selected monoclonal hybridoma cells were injected into BALB/c mice to produce ascites. The ascites were purified via ammonium sulfate precipitation, dialyzed against 0.01 M PBS to obtain a monoclonal antibody, and stored at −20 °C after freeze-drying.

The isotype of the obtained monoclonal antibody was detected using a mouse monoclonal antibody isotyping kit. The sensitivity (50% inhibitory concentration, IC50) and limit of detection (20% inhibitory concentration, IC20) were calculated from a standard curve. The specificity of the monoclonal antibody was determined by calculating the cross-reactivity (CR) of the monoclonal antibody to five other sulfonylurea herbicides. CR was calculated as the IC50 of HM IC50 of analogs.

### 2.5. Establishment and Optimization of the dcELISA

**Figure 2:**
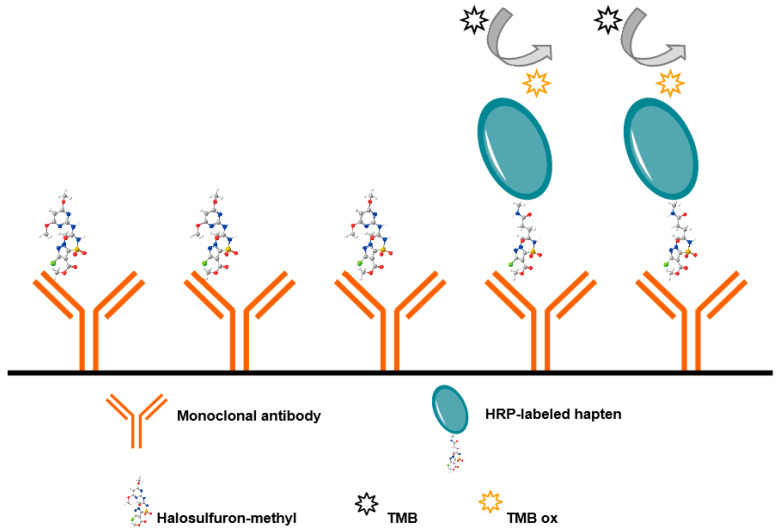
Schematic of dcELISA.

The direct and competitive ELISAs were developed using the above-described monoclonal antibody and the coating antigen HRP-hapten conjugate. The format of dcELISA is shown in Figure 2. Briefly, polystyrene microplate wells were coated with a monoclonal antibody in PBS (pH = 7.4, 100 μL/well) for 3 h at 4 °C; then, the plate was washed three times with PBSTG. The HM standard (50 μL/well) and HRP-hapten conjugate (50 μL/well) were diluted in PBSTG and added into wells sequentially. The plate was incubated for 15 min at 4 °C and washed three times with PBSTG. After passing the plate three times, the substrate solution was added, and the reaction was stopped as mentioned for the icELISA above.

The buffer pH, ionic strength, and organic solvent were optimized for the dcELISA. The sample diluting buffer was evaluated from pH = 5.5 to pH = 9.5. The ionic strength effect on the sample diluting buffer was estimated by changing the Na^+^ concentration from 0.05 M to 0.2 M. The organic solvent content effect was tested using different concentrations (5%, 10%, 15%, and 20%) of methanol and acetonitrile in sample diluting buffer, respectively. The results were evaluated based on the IC50 value and the coefficient of variation (*R*^2^) of the linear equation. The absorbance was measured at 490 nm on an Infinite M200 Pro microplate reader (Tecan Group Ltd., Männedorf, Switzerland).

### 2.6. Functionalization of MNPs with Antibodies

Conjugation of the magnetic nanoparticles with antibodies was performed according to a previous report [13]. In brief, a 50 μL aliquot of carboxyl magnetic beads (50 nm) was transferred to a “LoBind protein” microcentrifuge tube. Then, 50 μL of 10 mg/mL EDC and 150 μL of 10 mg/mL NHS were added. After mixing at room temperature for 20 min, the supernatant (containing PBST) was removed with a magnetic separator, and then the magnetic beads were resuspended in coupling buffer. For conjugation, 25 μg HM mAbs 1A91H11 were added to the magnetic beads, and the free antibodies were washed with PBST three times. After mixing for 2 h at ambient temperature, the MNPs/antibodies conjugation was blocked with blocking buffer for 20 min and washed with PBST three times. Finally, the precipitate was re-suspended in PBS solution (0.01 mol/L, pH = 7.4) and stored at 4 °C until use.

### 2.7. Establishment of the MLFIA

A mixture of 10 μL MNPs and 1 mL sample extract was added to a microcentrifuge tube and mixed at room temperature for 30 min. After removing the supernatant using the magnetic separator, the MNPs were redispersed in 100 μL 0.01 M PBS (pH = 7.4).

The lateral flow strip was assembled by sequentially pasting the adsorbent pad, nitrocellulose membrane, and sample pad onto a polyvinyl chloride (PVC) plate. The adsorbent pad and sample pad overlapped 1 and 2 mm with the nitrocellulose membrane, respectively. The nitrocellulose membrane was cut into 4 mm wide strips with a guillotine cutter. Then, the HM hapten-BSA conjugate and goat antimouse IgG were sprayed onto the nitrocellulose membrane to form test line (T) and a control line, respectively. Afterwards, the NC membrane with the test line and the control line was dried at 37 °C for 1 h.

### 2.8. Sample Extraction and Analysis

The extraction method refers to a reference with minor modifications [1]. The homogenized tomato sample (5 g) was weighed and extracted with 10 mL acetonitrile. After stirring at room temperature for 5 min, 1 g NaCl and 4 g anhydrous MgSO_4_ were added to the sample. The mixture was stirred for 3 min and then centrifuged at 5000 rpm for 5 min. The supernatant (1 mL) was further purified by adding 50 mg of C18 resin. After being centrifuged at 500 rpm for 5 min, the extract was dried under a nitrogen stream and resuspend by sample dilution buffer for further dcELISA analysis. For LC-MS/MS analysis, the extract was filtered through a 0.22 μM membrane and detected.

The paddy water (5 mL) was taken and filtered through a 0.22 μM membrane directly for MLFIA and LC-MS/MS analysis.

## 3. Results and Discussion

### 3.1. Characterization of the HM Hapten and Its Bioconjugates

The sulfonylurea bridge of HM is unstable, being readily hydrolyzed through bridge contraction and rearrangement under strong pH conditions [14], making synthesis of the hapten with the intact molecule infeasible. Simon et al. [23] and Zhao et al. [24] reported on the synthesis of haptens for metsulfuron-methyl and chlorimuron-ethyl, respectively, to produce antibodies. In those studies, the haptens contained half moieties of the herbicides and a succinic acid spacer arm that was conjugated to carrier protein, which was used to and immunize mice to produce high-affinity antibodies. We similarly designed a HM hapten and produced specific antibodies to HM (Figure 1A). By introducing a spacer arm through a C-N single bond at a sulfonamide position of 5-(aminosulfonyl)-3-chloro-1-methyl-1H-pyrazol moiety, this hapten was able to preserve the structure of HM with minimal alteration to the original framework.

Three HM bioconjugates, including hapten-BSA, hapten-OVA, and hapten-HRP, were prepared and used in the present study. First, the carboxylate group of the hapten was activated by N-hydroxysuccinimidyl ester, forming an active derivative. This active derivative reacted with the amino group on the carrier protein to form a bioconjugate, and the precise molar ratio (MR) of the hapten to protein was detected using MALDI-TOF-MS and calculated as follows: MR = (the mass of conjugate − the mass of protein)/mass of hapten. Mass spectra of the protein standards and bioconjugates are shown in Figure S3. The MRs were estimated to be 7:1, 2:1, and 1:1 for BSA-hapten, OVA-hapten, and HRP-hapten, respectively.

### 3.2. Antibody Generation and Characterization

Novel monoclonal antibodies for HM were produced from mice immunized with the BSA-hapten conjugate. The mouse with the highest titer and the best inhibition to HM in checkerboard icELISA was used for screening monoclonal hybridoma cells. After cloning, a limiting dilution assay was used to identify a positive monoclonal hybridoma, clone 1A91H11, that secreted antibodies against HM. Clone 1A91H11 was then cultivated and used to produce monoclonal antibodies. According to a checkerboard titration, the optimal dilution factor of the coating antigen OVA-hapten conjugate (1 mg/mL) and monoclonal antibody 1A91H11 (1 mg/mL) was 1.6 × 10^5^ times for icELISA. Figure S4 shows the standard inhibition curve of HM. The IC50 value and detection range were 16.5 ng/mL and 8.1 ng/mL–44.9 ng/mL (inhibition rate between 20–80%).

To evaluate the specificity of monoclonal antibody 1A91H11, cross-reactivity (CR%) of the antibody with several sulfonyl ureas, including 5-(aminosulfonyl)-3-chloro-1-methyl-1H-pyrazol, pyrazosulfuron-ethyl, nicosulfuron, chlorsulfuron, ethoxysulfuron, and chlorimuron-ethyl, were evaluated using icELISA. Except for the 0.06% CR with 5-(aminosulfonyl)-3-chloro-1-methyl-1H- pyrazol, MAb 1A91H11 showed no cross-reactivity with the tested sulfonylurea herbicides (Table 1). This indicated the validity of the novel hapten and the acceptable specificity of the antibody for accurate HM analysis.

### 3.3. Optimization of dcELISA Conditions

**Figure 3:**
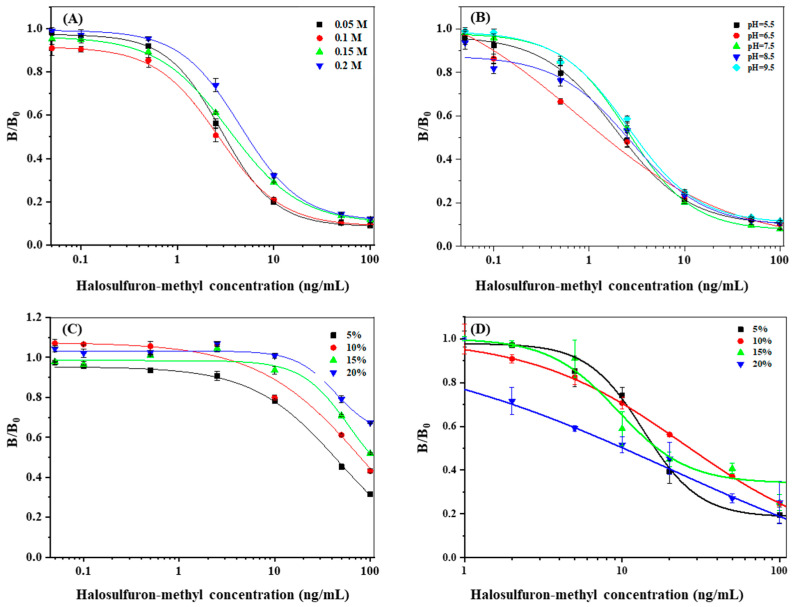
Optimization of dcELISA: (**A**) pH; (**B**) Na^+^ concentration; (**C**) Methanol concentration; (**D**) Acetonitrile concentration.

dcELISA is an immobilized antibody assay based on the competition between an enzyme-labeled hapten and an unknown amount of analyte for the immobilized antibody. dcELSIA is more sensitive and time-saving than icELISA [25]. To optimize the HM dcELISA, influencing factors, such as the optimal ratio of coating monoclonal antibody and hapten-HRP, pH value, and the diluting buffer NaCl concentration, were determined. According to the checkerboard dcELISA results, the dilution ratios of coating monoclonal antibody 1A91H11 (1 mg/mL) and hapten-HRP conjugate (1 mg/mL) were 1:200 and 1:3200. As shown in Figure 3, among the tested diluting buffer pH values (5.5, 6.5, 7.5, 8.5, and 9.5), the lowest IC50 (1.5 ng/mL) was achieved at pH 6.5. The ionic strength had little effect on the IC50 of the dcELISA when the NaCl concentration ranged from 0.05 to 0.2 M. When the diluting buffer contained 5% methanol (*v*/*v*), the sensitivity of dcELISA significantly decreased (IC50 42.5 ng/mL), most likely due to inhibition of the HRP-labeled hapten and the interaction between the antigen and antibody. Thus, methanol should be avoided in the extraction. As the acetonitrile concentration was increased from 5% to 20%, the IC50 values were observed to fall within the range of 10.15 ng/mL–26.91 ng/mL, indicating a minimal influence of acetonitrile on the dcELISA. This suggests that acetonitrile can be considered as a suitable extraction solvent when using the dcELISA method for sample detection.

**Figure 4:**
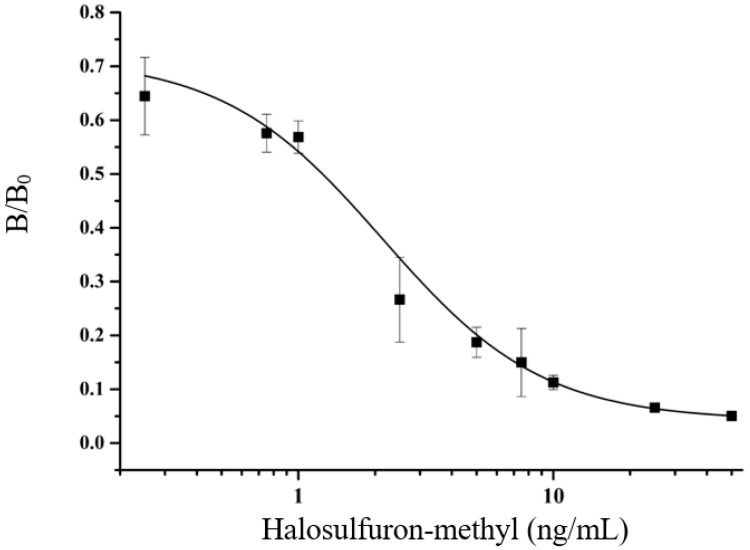
dcELISA calibration curve of HM.

The inhibition curve of dcELISA was obtained under the determined optimal conditions, pH 6.5, 0.01 M NaCl, and without methanol, resulting in an IC50 value of 1.5 × 10^−3^ mg/kg and working range of 0.7 × 10^−3^ mg/kg–10.7 × 10^−3^ mg/kg (inhibition rate between 20–80%) as shown in Figure 4. The sensitivity (IC50 = 1.5 × 10^−3^ mg/kg) of the dcELISA method for detecting HM residues was lower than the LOD of 2 × 10^−3^ mg/kg achieved by the CE-MS/MS method and similar to the LOQ of 1 × 10^−3^ mg/kg reported for the LC-MS/MS method [2,3].

### 3.4. Optimization of the MLFIA

**Figure 5:**
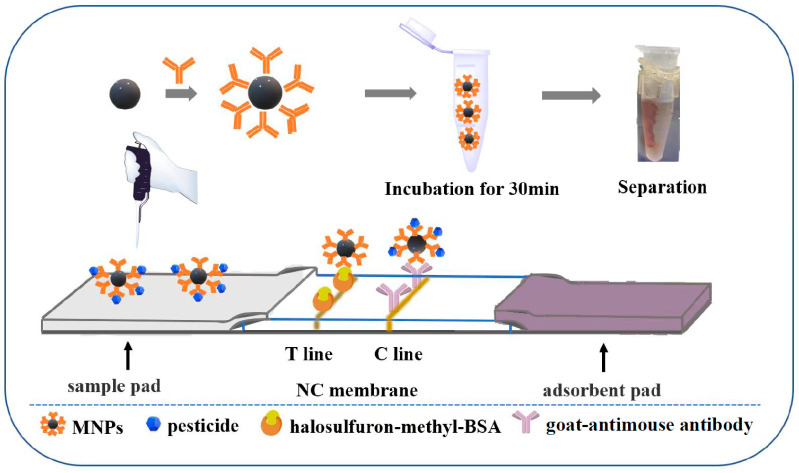
Illustration of a magnetic lateral flow immunoassay.

The ratio of EDC to NHS, amount of HM antibodies, pH of the magnetic beads–antibodies coupling reaction, and the concentrations of hapten-BSA and goat–antimouse antibody applied to the NC membrane were systematically investigated to improve the sensitivity of the MLFIA. The format of MLFIA is shown in Figure 5.

#### 3.4.1. The EDC/NHS Ratio

**Figure 6:**
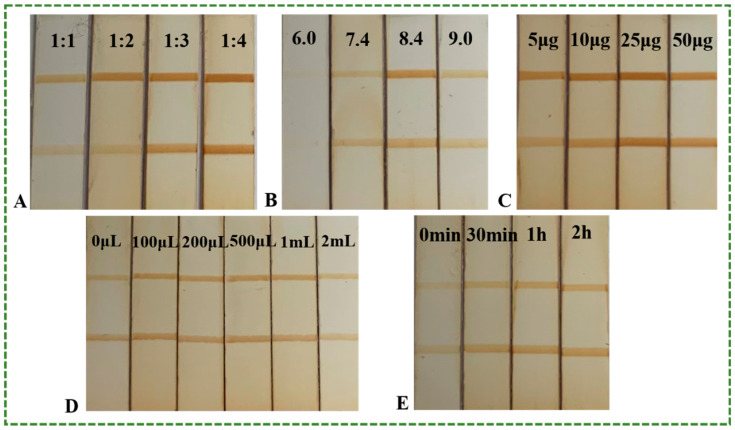
Optimization of MLFIA: (**A**) EDC/NHS ratio; (**B**) pH value; (**C**) Antibody amount; (**D**) Enrichment volume; (**E**) Enrichment time.

Complementary interactions between EDC and NHS can increase the stability and yield of the conjugation reaction. Therefore, different ratios of EDC/NHS (from 1:1 to 1:4) were evaluated. As shown in Figure 6, the colors of both the T and C lines deepened as the EDC/NHS ratio increased (Figure 6A). However, no obvious difference was observed between 1:3 and 1:4. On the basis of our experimental results, 1:3 was chosen as the EDC/NHS ratio for the MLFIA.

#### 3.4.2. HM Antibodies Content and pH of the Conjugation Solution

Four different amounts of HM antibody 1A91H11 (5 μg, 10 μg, 25 μg, and 50 μg) were tested. As shown in Figure 6C, the darkest of T and C lines were obtained when 25 μg were added. Considering the pH can affect the coupling efficiency, the influence of pH from 6.0 to 9.0 was evaluated. The darkest T and C lines were obtained at pH 8.4 (Figure 6B). Therefore, 25 μg of HM antibody and pH 8.4 were selected as the optimal conditions of the conjugation solution.

#### 3.4.3. Optimization of MNP Probe Enrichment Time and Volume

When added to LFIA, MNPs improve the detection limit by acting as color reagents and by providing an enrichment effect. To obtain better immunoassay performance, MNP probe enrichment time and volume were optimized. Ten μL MNP probes were added into 10 ng/mL HM at different volumes (100 μL, 200 μL, 500 μL, 1 mL, and 2 mL). As displayed in Figure 6D, the colors of both the T and C lines deepened with the addition of 200 μL, remained the same from 200 μL to 1 mL, and lightened at 2 mL. Subsequently, the mixture of MNP probes and HM standard were stirred for 15 min, 30 min, 1 h, and 2 h. As revealed in Figure 6E, colors of both the T and C lines increased from 15 min to 30 min and remained the same after 30 min. Therefore, 1 mL and 30 min were chosen as the optimal enrichment volume and time for the following experiments.

### 3.5. Evaluation and Specificity of MLFIA and dcELISA

**Figure 7:**
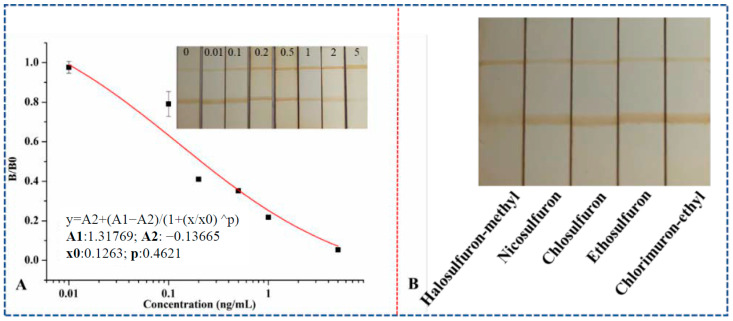
Standard curve for HM analysis (**A**) and specificity results (**B**) of MLFIA.

Compared with dcELISA (IC50 1.5 ng/mL), MLFIA has the advantage of high sensitivity due to the enrichment effect and reduction of matrix effects. Serial dilutions of the extraction solution for MLFIA ranging from 0.01 to 5 ng/mL were tested, and standard curves of the MLFIA for HM were established under the optimal assay conditions (Figure 7A). The assay had an IC50 value of 0.21 ng/mL and good linear fitting (*R*^2^ ≥ 0.95) with concentrations ranging from 0.03 to 1.5 ng/mL (inhibition rate between 20 and 80%).

To evaluate the specificity of MLFIA for the detection of HM, 2 ng/mL of HM and 20 ng/mL of pyrazosulfuron-ethyl, nicosulfuron, chlorsulfuron, ethoxysulfuron, and chlorimuron-ethyl standard solutions were detected using MLFIA, in which the color intensity of T and C lines was measured using a test strip reader. The results are shown in Figure 7B. The T/C values of the blank solution and the analog standards were higher than the HM standard, suggesting good selectivity of the MLFIA, which was consistent with the mAb specificity.

### 3.6. Food Sample Analysis

HM is widely registered for use in tomato and maize cultivation in China, which poses the risk that its residue could be present in those foods. Tomato and maize samples were spiked with 0.025 mg/kg, 0.05 mg/kg and 0.1 mg/kg HM. The average recoveries were detected using dcELISA and LC-MS/MS. According to results shown in Table 2, the average recoveries of HM using dcELISA were 78.9–87.9% and 103.0–107.4% in tomato and maize with RSDs of 1.1–6.8% and 2.7–6.4%, respectively. The average recoveries of HM using LC-MS were 77.2–87.6% and 92.1–93.0% in tomato and maize with RSDs of 1.2–2.6% and 6.0–7.7%, respectively. The results of dcELISA were in agreement with those detected using LCMS/MS, which strengthens the reliability of dcELISA for the analysis of HM residue in tomato and maize matrices.

Similarly, paddy water was spiked with 0.025, 0.05, and 0.1 mg/kg HM standard. The results are presented in Table 3. The average recoveries and RSDs of the LC-MS/MS ranged from 93.5 to 98.9% and 1.8 to 2.7%, while the average recoveries and RSDs of the developed MLFIA ranged from 81.5 to 92.5% and 5.4 to 9.7%, which generally satisfied the requirements of trace detection. Regarding paddy water, the developed MLFIA showed acceptable average recoveries and RSDs. The developed MLFIA method may be suitable for the rapid analysis of HM residue in paddy water samples.

## 4. Conclusions

In the present research, the design of a novel HM hapten followed a similar method to that of other sulfonylurea herbicides, such as triasulfuron [11], chlorsulfuron [26], and chlorimuron [13]; i.e., one step synthesis of the hapten was carried out with the half moiety of HM. Unlike other studies, the present hapten introduced a carboxyl group to the pyrazole-sulfonamide moiety of halosulfuron with a four-carbon length, which enabled sufficient exposure of the carrier-protein-attached hapten to the immune system [10]. Due to this, the resulting Mab 1A91H11 was highly sensitive to the target analyte. A highly sensitive dcELISA using Mab 1A91H11 and hapten-labeled HRP was developed, optimized, and validated for the determination of HM in tomato and maize samples. The dcELISA developed here, with an IC50 value of 1.5 ng/mL, was more convenient and 10 times more sensitive than the typically employed indirect competitive assay to detect small molecules. This dcELISA was optimized to be a rapid and sensitive alternative tool to detect HM in tomato and maize samples. For the convenience of field detection, therefore, a magnetic-beads-based immunoassay with higher sensitivity and shorter detection time was developed. Magnetic beads played the role of concentration, enrichment, and signal detection. The MLFIA possessed an IC50 value of 0.21 ng/mL, which was 10 times lower than the dcELISA. MLFIA provides a development direction worthy of attention for rapid and highly sensitive detection of HM and other pesticide residues in food and the environment.

## Figures and Tables

**Table 1:** Cross reactivity of mAb 1A91H11 with HM and other sulfonylureas.

Analytes and Structures		IC50 (ng/mL)	Cross Reactivity
HM	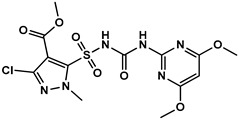	28.89	100%
HM hapten	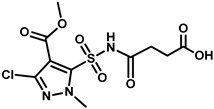	43.5 ^a^	0.06% ^a^
Nicosulfuron	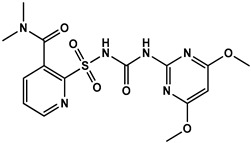	>20,000	<0.02%
Chlosulfuron	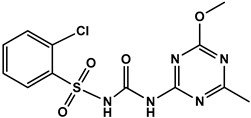	>20,000	<0.02%
Ethosulfuron	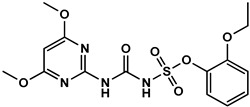	>20,000	<0.02%
Chlorimuron-ethyl	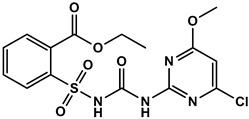	>20,000	<0.02%
Pyrazosulfuron-ethyl	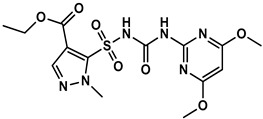	>20,000	<0.02%

^a^ μg/kg.

**Table 2:** Comparison between dcELISA and LC-MS/MS for halosulfuron recovery in tomato and maize.

Matrix	Spiked Halosulfuron Methyl (mg/kg)	Average Recovery and RSD (%)			
		dcELISA	RSD	LC-MS/MS	RSD
Tomato	0.025	78.9 ± 0.02	9.1	77.2 ± 0.9	1.2
	0.05	84.4 ± 0.01	1.1	87.2 ± 2.3	2.6
	0.1	87.9 ± 0.01	6.8	87.6 ± 1.3	1.8
Maize	0.025	107.4 ± 5.6	6.4	92.8 ± 8.2	6.0
	0.05	105.5 ± 7.3	7.7	93.0 ± 8.5	7.7
	0.1	103.0 ± 2.6	2.7	92.1 ± 6.0	7.6

**Table 3:** Comparison between MLFIA and LC-MS/MS analysis for halosulfuron recovery in paddy water.

Matrix	Spiked Halosulfuron Methyl (mg/kg)	Average Recovery and RSD (%)			
		MLFIA	RSD	LC-MS/MS	RSD
Paddy water	0.025	81.5	8.3	93.5	1.8
	0.05	92.5	9.7	98.9	2.3
	0.1	89.3	5.4	96.5	2.7

## Data Availability

The data used to support the findings of this study can be made available by the corresponding author upon request.

## Supplementary Materials

The following supporting information can be downloaded at https://www.mdpi.com/article/10.3390/foods12142764/s1. Figure S1: HRMS of the halosulfuron-methyl hapten. Figure S2: ^1^H NMR (a) and ^13^C NMR (b) spectra of the halosulfuron-methyl hapten. Figure S3: MALDI-TOF MS of halosulfuron-methyl antigens: (A) BSA vs. the BSA-hapten conjugate; (B) OVA vs. the OVA-hapten conjugate; (C) HRP vs. the HRP-hapten conjugate. Figure S4: The icELISA calibration curve of halosulfuron-methyl.
