# Supplementary material for: Development of Magnetic Lateral Flow and Direct Competitive Immunoassays for Sensitive and Specific Detection of Halosulfuron-Methyl Using a Novel Hapten and Monoclonal Antibody
Source: Foods. 2023 Jul 20;12(14):2764. doi: 10.3390/foods12142764 (PMC10378753; doi:10.3390/foods12142764)

Development of Magnetic Lateral Flow and Direct Competitive Immunoassays for Sensitive and Specific Detection of Halosulfuron-Methyl Using a Novel Hapten and Monoclonal Antibody

Ying Ying [1,2,†], Xueyan Cui [1,2,†], Hui Li [1,2], Lingyi Pan [1,2], Ting Luo [1,2], Zhen Cao [1,2,*] and Jing Wang [1,2]

[1] *Institute of Quality Standard and Testing Technology for Agro-Products, Key Laboratory of Agro-Product Quality and Safety, Chinese Academy of Agricultural Sciences, Key Laboratory of Agro-Product Quality and Safety, Ministry of Agriculture, Beijing 100081, China; yingying@caas.cn (Y.Y.); cxy19910411@163.com (X.C.); urhaan@163.com (L.P.); luoting074@163.com (T.L.); w_jing2001@126.com (J.W.)*

[2] *Institute of Quality Standards & Testing Technology for Agro-Products, Chinese Academy of Agricultural Sciences, Beijing 100081, China*

[*] *Correspondence: caozhen01@caas.cn*

[†] *These authors contributed equally to this work.*

*** Corresponding authors:**

Tel.: +86-10-8210-6513**. E-mail: c**aozhen@caas.com (Zh. Cao)

Supplementary Figure Captions

Figure S1. HRMS of the halosulfuron-methyl hapten.

Figure S2. ^{1}H NMR (a) and ^{13}C NMR (b) spectra of the halosulfuron-methyl hapten.

Figure S3. MALDI-TOF MS of halosulfuron-methyl antigens: (A) BSA vs. the BSA-hapten conjugate, (B) OVA vs. the OVA-hapten conjugate, and (C) HRP vs. the HRP-hapten conjugate.

Figure S4. The icELISA calibration curve of halosulfuron-methyl

Figure S1

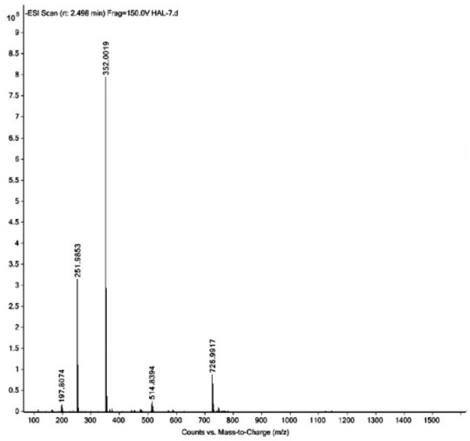

Figure S2

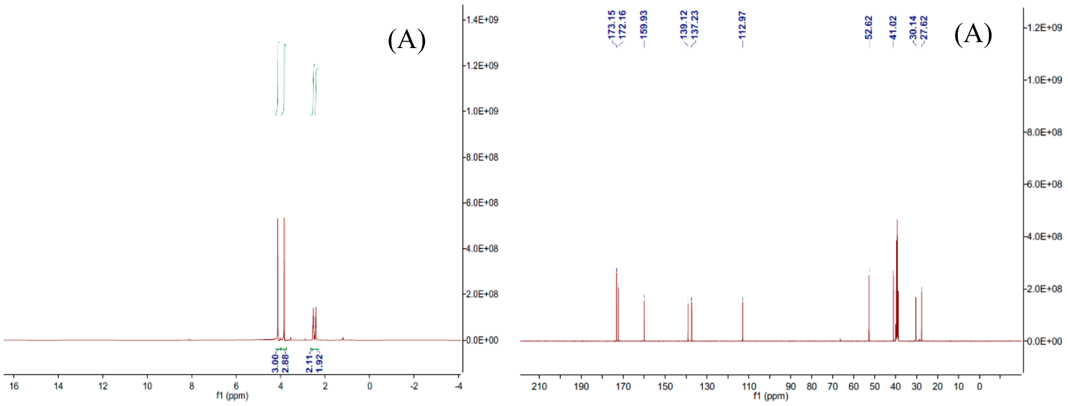

Figure S3

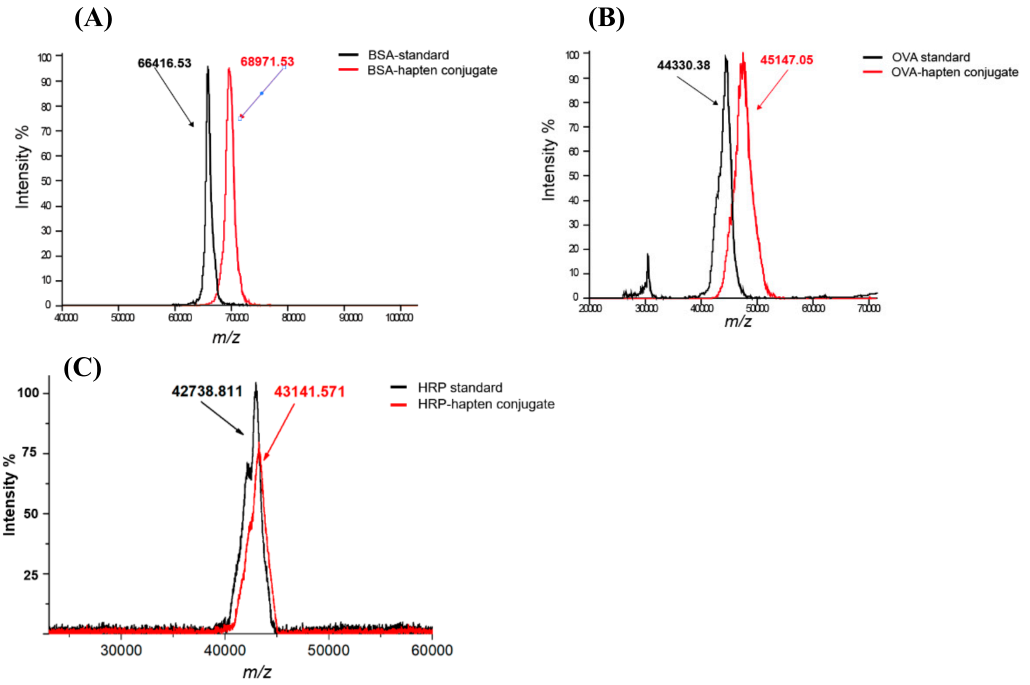

Figure S4

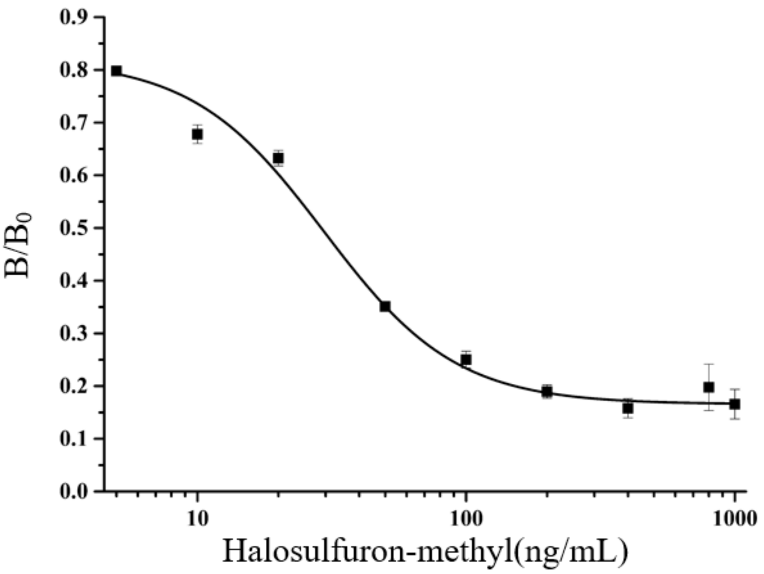

Supplement: Supplementary file 1 [file foods-12-02764-s001.zip › foods-2465645-supplementary.pdf]
